# Genome-scale CRISPRa screening identifies MTX1 as a contributor for sorafenib resistance in hepatocellular carcinoma by augmenting autophagy

**DOI:** 10.7150/ijbs.62393

**Published:** 2021-07-25

**Authors:** Li Li, Shijun Yu, Qingqing Hu, Yanan Hai, Yandong Li

**Affiliations:** Department of Oncology, Shanghai East Hospital, School of Medicine, Tongji University, Shanghai 200120, China

**Keywords:** HCC, MTX1, sorafenib, autophagy, CISD1

## Abstract

Sorafenib is the standard first-line drug for the treatment of advanced hepatocellular carcinoma (HCC), however, its therapeutic efficacy is not satisfactory due to primary or secondary resistance of HCC cells. In the present study, we identified Metaxin 1 (MTX1) as a new regulator of sorafenib resistance in HCC through genome-scale CRISPR activation (CRISPRa) screening. We found that MTX1 was frequently upregulated in HCC tissues and overexpression of MTX1 promoted HCC cell proliferation *in vitro* and* in vivo*. As well, MTX1 overexpression increased cell growth rate and decreased cell apoptosis upon sorafenib treatment. Consistently, the resistance induced by MTX1 was also observed in subcutaneous xenograft tumor model. Clinically, high expression of MTX1 was closely related with poor outcomes in HCC patients who received sorafenib treatment. Mechanistically, overexpression of MTX1 could promote HCC cell autophagy via interacting with and inhibiting CDGSH iron sulfur domain 1 (CISD1), an autophagy negative regulator. Taken together, our findings suggest that MTX1 is upregulated in HCC and contributes to sorafenib resistance via a possible mechanism involving CISD1 mediated autophagy.

## Introduction

Hepatocellular carcinoma (HCC) is one of the most common malignancies and the third cause of cancer-associated mortality worldwide 1. Sorafenib, an oral multi-kinase inhibitor, has become a mainstay of standard treatment for advanced HCC patients since 2007 2. Its multi-kinase targets include RAF proto-oncogene (RAF), vascular endothelial growth factor (VEGF), platelet-derived growth factor (PDGF), FMS-like tyrosine kinase 3 (FLT3) and KIT proto-oncogene (KIT) 3, 4. The inhibition of these kinases affects HCC cell growth, proliferation or survival. Nevertheless, the clinical efficacy of sorafenib is frequently limited by drug resistance in a considerable number of HCC patients 5, 6. Therefore, understanding the molecular mechanism of sorafenib resistance and searching for effective biomarkers are crucial for predicting or overcoming sorafenib resistance for HCC patients.

The clustered regularly interspaced short palindromic repeats/CRISPR-associated (CRISPR/Cas) system has been rapidly developed as a powerful tool for gene editing in recent years 7. The system has currently innovated CRISPR interference (CRISPRi) or CRISPR activation (CRISPRa) library, which can quickly knock out or amplify any genes in the human genome 8, 9. The genome-wide screening is an effective and unbiased approach to determine genes or pathways involved in biological processes 10. To date, several studies have applied the technique to successfully pick out the required genes or pathways. For example, kinases that regulate response to fibroblast growth factor receptor (FGFR) inhibitor have been found in gastric cancer 11, and the Wnt-FZD5 signaling pathway that regulates drug sensitivity has been identified in RNF43-mutant pancreatic tumors 12. In the present work, in order to identify genes which are essential to sorafenib resistance in HCC, we conducted CRISPR-Pool™ SAM human library comprising 70,290 SAM-sgRNAs upon HCC cells to amplify genes for screening purposes. Among the potential sorafenib resistance factors, Metaxin 1 (MTX1) was considered as the most effective druggable target genes.

MTX1 belongs to Metaxins family, which also includes another member Metaxin 2 (MTX2). As the outer membrane proteins of mitochondria, the Metaxins family participates in the import of nuclear-encoded proteins into mitochondria and is thought to be the member of Sorting and Assembly Machinery (SAM) 13, 14. Some studies reveal that MTX1 is essential for the proapoptotic protein Bak (BCL2 antagonist/killer 1) activation during tumor necrosis factor (TNFα)-induced cell death 14, 15. In addition, genome-wide association studies (GWASs) have reported that MTX1 is one of the high-quality biomarkers of gastric cancer susceptibility 16, implicating the key role of MTX1 in promoting cancer progression. However, the precise biological function of MTX1 in HCC remains unclear.

In the present study, we identified MTX1 as a new regulatory factor involving sorafenib resistance by CRISPR/Cas9 activation library screening. Further investigations indicated that MTX1 was upregulated in HCC tissues and enhanced sorafenib resistance of HCC cells *in vitro* and *in vivo*. Finally, CISD1 was found to be interacted with MIX1, which may antagonize MTX1-caused autophagy during sorafenib treatment.

## Materials and methods

### Human HCC tissue specimens

Forty paired tumor and corresponding normal liver samples derived from HCC patients were collected from Shanghai East Hospital in Shanghai, China. Following approval by the Ethics Committee of Shanghai East Hospital, each patient signed an informed consent before participating in the study. All fresh tissues were preserved in liquid nitrogen before use.

### Cell culture, reagents, and antibodies

Human HCC cell lines PLC/PRF/5 and Huh7 were purchased from the Type Culture Collection of the Chinese Academy of Sciences, Shanghai, China. HCC-LM3 cells were taken from our laboratory stocks. All the HCC cells were cultured in Dulbecco's modified Eagle's medium (DMEM; Corning, Inc., Corning, NY, USA) with 10% fetal bovine serum (FBS) and 1% penicillin/streptomycin, and were maintained in an incubator with 5% CO_2_ at 37 °C. Sorafenib and Bafilomycin A1 were obtained from Selleck Chemicals (Houston, TX, USA). Primary antibodies used in this study were listed below: MTX1 (15529-1-AP, Proteintech, Wuhan, China), CISD1 (16006-1-AP, Proteintech, Wuhan, China), Beclin1 (11306-1-AP, Proteintech, Wuhan, China), LC3 (14600-1-AP, Proteintech, Wuhan, China), GAPDH (60004-1-Ig, Proteintech, Wuhan, China), β-Actin (sc-8432, Santa Cruz Biotechnology, USA) and FLAG (abs830005, Absin, China).

### Lenti CRISPR/cas9 screening and sequencing

CRISPR-Pool™SAM-Human-Dual vector was purchased from Shanghai Genechem Co., Ltd (Shanghai, China). The CRISPR/Cas9 synergistic activator (SAM) system can activate gene transcription by using dCas9-VP64 to achieve the upregulation of coding genes. More specifically, HCC-LM3 cells were transduced with EF1α-MS2-p65-HSF1-2A-Hygro-WPRE lentivirus, and the successfully infected cells were screened with Hygromycin B (200 μg/ml). Next, the cells were infected with CRISPR-Pool™SAM human/Dual lentivirus. Two days later, Blasticidin S (10 μg/ml) and Hygromycin B (100 μg/ml) were added to the medium to select stably transduced cells. We classified the surviving cells into control or experimental group. For experimental group, the cells were continuously treated with sorafenib (7.5 μM) for 14 days. The viable cells from control or experimental group respectively were harvested to extract genomic DNA using DNA extraction kit (DP304, Tiangen Biotech, Beijing, China). And genomic DNA was performed to amply sgRNA targeted region using PCR technology. The enrichments of sgRNAs were identified by next generation sequencing (Oebiotech, Shanghai, China).

### RNA interference and gene overexpression

Short-interfering RNAs (siRNAs) targeting MTX1 or CISD1 were synthesized by Shanghai Genepharma Co., Ltd, Shanghai, China. The sequences were as follows: siNC: 5′-UUCUCCGAACGUGUCACGU-3′; siMTX1: 5'-CCCACAUUCUCAGUCUCUA-3'; siCISD1-1: 5'-CAGCUGCAAUUGGUUAUCU-3'; siCISD1-2: 5'-CGUGAAGUUACCUGAUUGU-3'.

For gene overexpression, FLAG-tagged MTX1 plasmid was constructed through cloning MTX1 cDNA (GeneBank Accession Number: NM_002455.5) into pcDNA3.1 vector. CISD1 overexpression plasmid (also FLAG tagged) was constructed based on pENTER vector, which was obtained from Vigene Biosciences, Shandong, China. MTX1-overexpressing lentivirus was prepared by Shanghai Genepharma Co., Ltd, Shanghai, China.

### Cell proliferation assays

Cell growth ability was measured by Cell Counting Kit-8 (CCK-8, Dojindo Laboratories, Japan) according to the manufacturer's instructions. Briefly, cells were seeded into 96-well plates with a density of 3×10^3^ cells per well in triplicate, and absorbance was read at 450 nm by spectrophotometer. For the clonogenic assay, cells (1×10^3^ cells /well) were plated in 6-well plates and incubated for 14 days, and cell culture medium was replaced every 3 days. The colonies were immobilized with 4% paraformaldehyde and then stained with crystal violet. Each assay was repeated at least three times.

### Flow cytometry

An Annexin V, FITC apoptosis detection kit (Dojindo Laboratories, Japan) was utilized to measure cell apoptotic ratio. HCC cells treated or untreated with sorafenib for 48 h were trypsinized, centrifuged, washed with cold PBS according to the protocol. The cells were then resuspended with 1× Annexin V Binding Solution, and Annexin V, FITC and PI solutions were added to the cell suspension. Cell apoptosis rate was detected by flow cytometry after the cells were incubated in the dark at room temperature for 15 minutes. Results were analyzed using FlowJo software (FlowJo, LLC). Each assay was repeated at least three times.

### Western blot assay

Whole cell lysates were extracted using RIPA buffer (#20-188, Merck Millipore, MA, USA) supplemented with protease inhibitor cocktail (Sigma-Aldrich; Merck KGaA, Darmstadt, Germany), Total protein was separated by SDS-PAGE and transferred onto a nitrocellulose filter membrane. Then, the membrane was blocked in 5% non-fat milk for 1 h at room temperature. After primary and secondary antibodies incubation, signals were visualized using the Odyssey Infrared imaging system (LI-COR Biosciences, Lincoln, NE, USA).

### Quantitative real-time PCR (qRT-PCR)

Total RNA was harvested from cells or tissues using Trizol (Invitrogen, USA) and reverse transcribed into cDNA using PrimeScript™ RT reagent kit (Takara Bio, Japan). Real-Time PCR was conducted with TB Green® Premix Ex Taq™ (Tli RNaseH Plus) kit (Takara Bio, Japan) according to the manufacturer's protocol. Using human β-actin gene as the internal control, the relative mRNA level of target genes was determined by 2^-ΔΔCT^ method. Each assay was repeated at least three times. The primers used in this experiment were as follows:

MTX1-F: 5'-GAAGTGACCCGGAAGTGGTAT-3';

MTX1-R: 5'-GTAGCCGTTCCATGTACTGCC-3';

β-actin-F: 5'-AGAGCCTCGCCTTTGCCGATCC-3';

β-actin-R: 5'-CTGGGCCTCGTCGCCCACATA-3'.

### Mouse model

Athymic nude mice aged 6 weeks were purchased from SIPPR-BK Lab Animal Co., Ltd, Shanghai, China to establish a xenograft model of HCC. HCC-LM3 and Huh7 cells were stably infected with lentivirus overexpressing MTX1 or their control, and subcutaneously injected into each mouse. The size of tumor was recorded every week. Tumor volume was calculated according to the formula: Tumor volume = (Length × Width^ 2^)/2. Four weeks later, the mice were sacrificed, and the tumors were collected and weighted. For Huh7 cells, above mice were once more randomly divided into two groups: sorafenib-untreated or treated groups. Sorafenib was administered at the dose of 80 mg/kg by oral gavage every other day. After 3 weeks of sorafenib treatment, the mice were euthanized and the tumors were resected, weighed and photographed. All animal experiments have been approved by the Ethics Committee of Shanghai East Hospital, and are carried out strictly according to the guidelines.

### Bioinformatics analysis

In this work, we used bioinformatics softwares and websites to obtain broader evidence. LENEP, BCL11B, BAG3, RANBP1, and MTX1 mRNA expression levels were analyzed using GEPIA (http://gepia.cancer-pku.cn/). The relationship between gene expression and survival was analyzed by UALCAN (http://ualcan.path.uab.edu/analysis.html). Additionally, geneMANIA (http://genemania.org/) predicted the interaction of MTX1 and CISD1.

### Autophagy analysis by LC3 monitoring

In short, cells with stable expression of MTX1 or control were transfected with the mCherry-hLC3B-pcDNA3.1 plasmid, and then were treated with sorafenib for 24 h. The level of autophagy was manifested as the number of LC3 puncta, which was observed by fluorescence microscope. The mCherry-hLC3B-pcDNA3.1 plasmid was a gift from David Rubinsztein (Addgene plasmid # 40827) 17. Each assay was repeated at least three times.

### Electron microscopy

Cells were immobilized with 2.5% glutaraldehyde with 0.1 M sodium cacodylate. The sample was dehydrated with a series of gradient ethanol and embedded in an epoxy resin. After staining with 3% uranyl acetate and lead citrate, the sample was observed under a transmission electron microscope. Each assay was repeated at least three times.

### Tissue microarray

A tissue microarray (Cat # LVC1607) consisting of 80 pairs of primary HCC tissues and corresponding non-tumor tissues was purchased from Outdo Biotech Company (Shanghai, China). The tissue samples were derived from HCC patients who received sorafenib treatment after surgical resection. MTX1 antibody (15529-1-AP, Proteintech, Wuhan, China) was used to examine MTX1 expression in the HCC tissue microarray. The scoring criteria of immunohistochemical staining were completed by two pathologists in Shanghai East Hospital through evaluating the intensity and percentage of MTX1-positive staining. The staining of MTX1 was classified as negative, weak or strong. Additionally, negative and weak staining was considered as MTX1 low expression level, and strong staining was identified as MTX1 high expression level.

### Co-immunoprecipitation (Co-IP)

Immunoprecipitation was performed as previously described 18. Briefly, total proteins were extracted using IP lysis buffer (20mM Tris, pH7.5; 150mM NaCl; 1.0% Triton X-100; 1mM ethylenediaminetetraacetic acid; and protease inhibitor cocktail). Cell supernatants were collected and mixed with FLAG antibody or CISD1 antibody at 4 °C for 2 h, and then Dynabeads Protein G (Invitrogen) were added overnight at 4 °C. The beads were washed 4 times with IP lysis buffer, resuspended in 2x SDS loading buffer (#KGP101X, Keygentec Inc., Shanghai, China), and the interaction was finally analyzed by western blot analysis. Each assay was repeated at least three times.

### Statistical analysis

All data were presented as the mean ± standard deviation, unless stated otherwise. A two-tailed Student's t-test was used to compare the differences between the two groups. Comparisons among more than three groups were carried out by one-way ANOVA. Survival curve was performed to analyze the survival probability. *P* < 0.05 was considered statistically significance. All statistical analyses were performed by GraphPad Prism 7 (GraphPad Software, Inc.).

## Results

### Genome-scale CRISPRa screening identifies genes involved in sorafenib resistance in HCC cells

**Figure 1:**
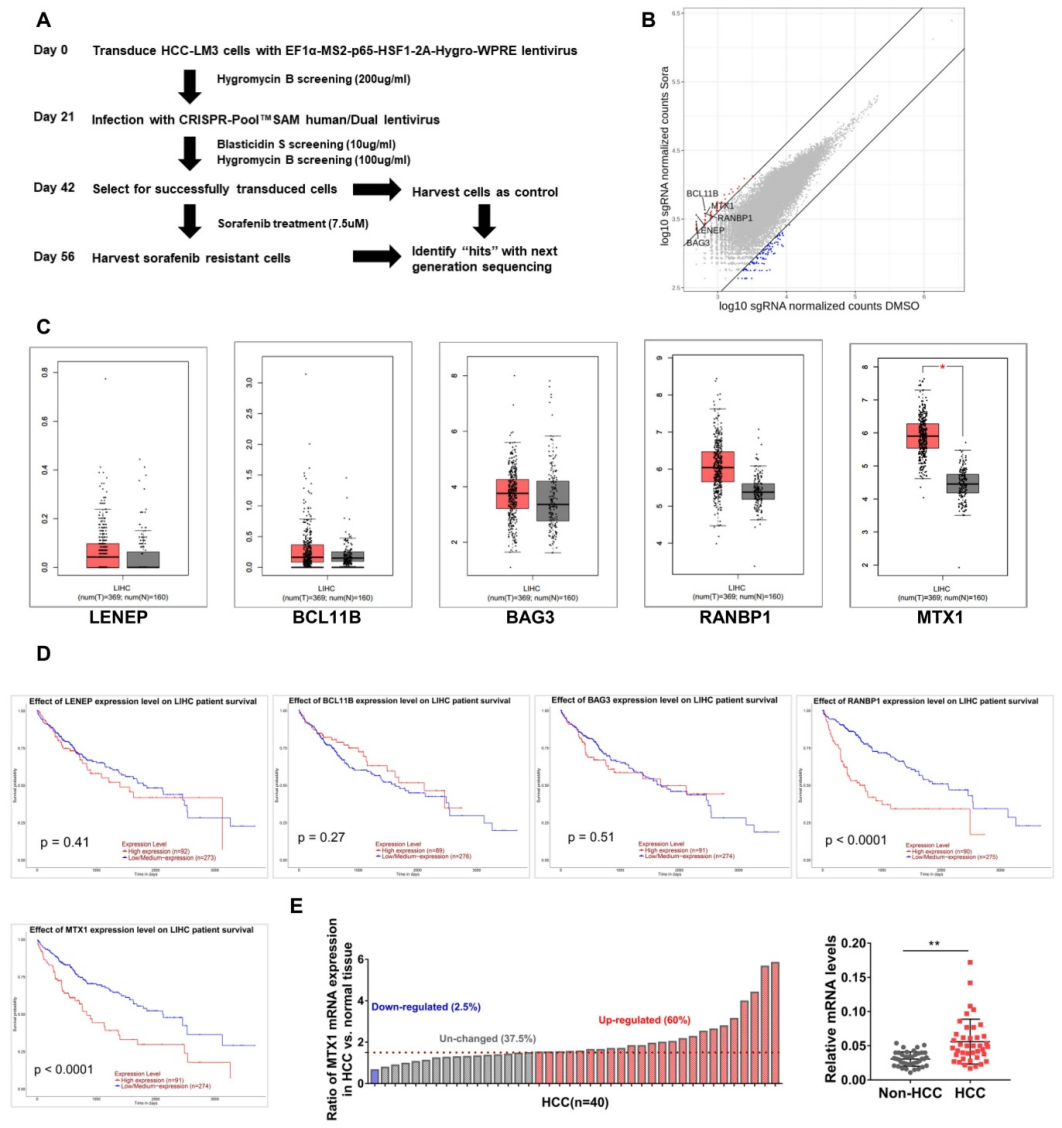
** MTX1 was identified as a sorafenib resistance factor by Genome-Scale CRISPRa Screening. (A)** Flow chart of CRISPR-Pool™SAM human library screening for genes involving in sorafenib resistance in HCC-LM3 cells. **(B)** Scatterplot displayed enrichment of sgRNAs in surviving cells after sorafenib treatment compared to the control group. **(C)** Expression boxplot of LENEP, BCL11B, BAG3, RANBP1, and MTX1 in human HCC patients from GEPIA online database (http://gepia.cancer-pku.cn/). Red boxes indicate tumor tissue and gray boxes represent normal tissue. **(D)** Kaplan-Meier survival curves were analyzed for above candidate genes based on UALCAN (http://ualcan.path.uab.edu/analysis.html). **(E)** The mRNA expression level of MTX1 in 40 pairs of HCC and adjacent normal samples were determined by qRT-PCR. The data of qRT-PCR are presented as mean ± SD from three experiments, **P < 0.01.

In order to explore those genes involved in sorafenib resistance of HCC, a genetic screening was conducted upon HCC-LM3 cells using the CRISPR-Pool™SAM human library, which contains 70,290 SAM-sgRNA targets including 23,430 coding gene transcripts. The screening procedure was shown in Figure 1A. Compared with controls, the enrichment of sgRNAs in 2456 genes was altered after sorafenib treatment. These genes were ranked based on the fold change of sorafenib/control group, as demonstrated in Figure 1B, and the top 5 genes were lens epithelial protein (LENEP), BAF chromatin remodeling complex subunit BCL11B (BCL11B), BAG cochaperone 3 (BAG3), RAN binding protein 1 (RANBP1), and metaxin 1 (MTX1). To understand the putative effects of these genes in HCC progression, we compared their expression between HCC samples and normal tissues in TCGA database through GEPIA website (Figure 1C). As well, we analyzed the prognosis significance of these genes through UALCAN online tools (Figure 1D). Among these genes, remarkable overexpression of MTX1 was found in HCC specimens and high expression of MTX1 was associated with poor survival of HCC patients. In addition, our qRT-PCR data confirmed the upregulation of MTX1 in HCC tumor tissues from our hospital (Figure 1E). These results implied that MTX1 might be a sorafenib resistance factor and contribute to HCC development.

### Enforced expression of MTX1 could accelerate HCC cell proliferation

**Figure 2:**
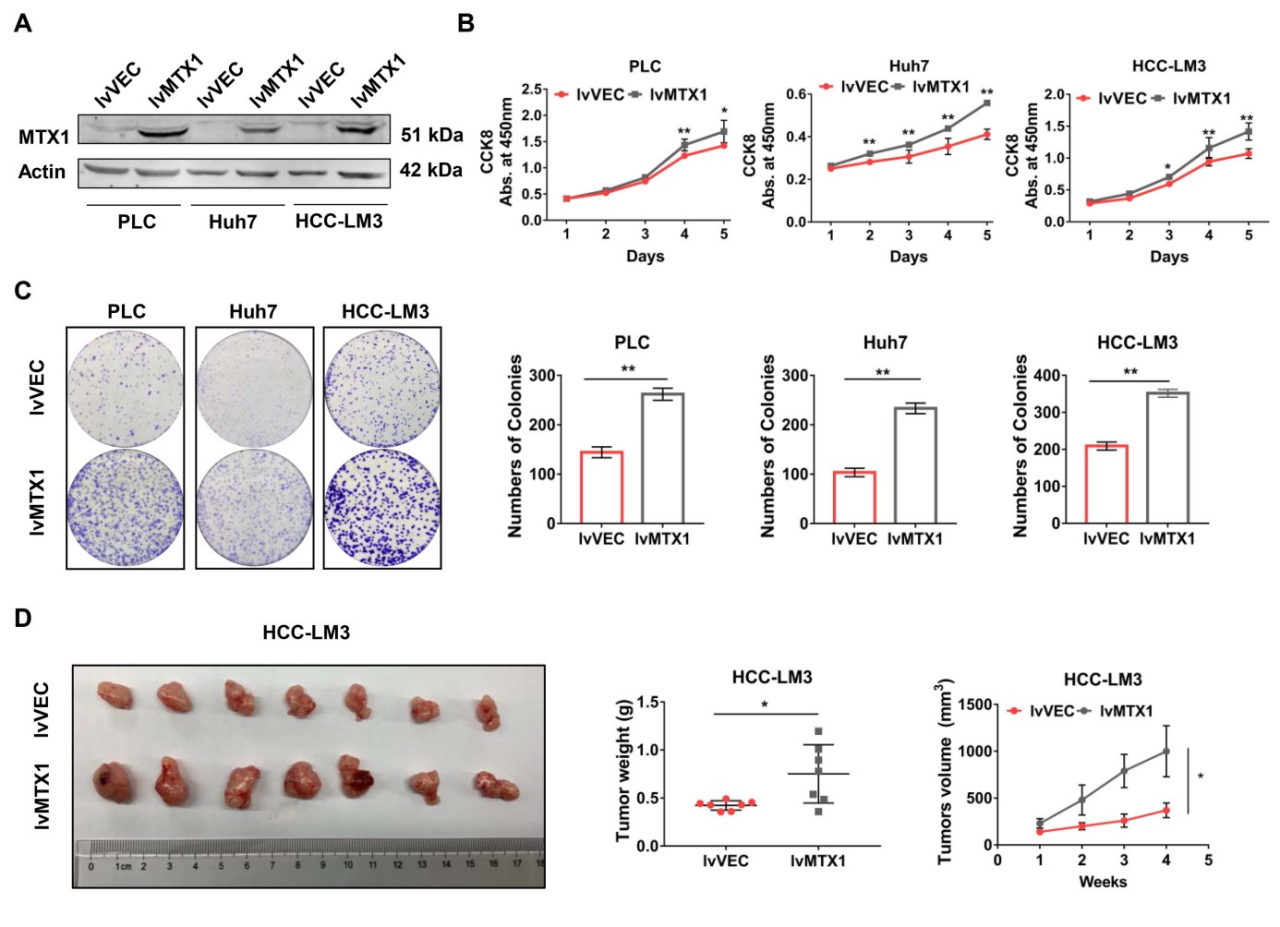
**Enforced expression of MTX1 accelerated HCC cell growth. (A)** Overexpression of MTX1 was confirmed by western blot. **(B)** CCK-8 assays were performed using cells with or without stable MTX1 overexpression, and cell growth curves were shown. **(C)** Effects of upregulated MTX1 expression on cell proliferation were validated by the clonogenic assay. **(D)**. Forced expression of MTX1 increased xenograft tumor growth in nude mice. Tumor weights were measured after the mice were killed. Tumor volumes were recorded every week. All of results are shown as mean ± SD from at least three experiments, *P < 0.05, **P < 0.01.

To examine the functional role of MTX1 in HCC progression, gain-of-function experiments were performed in three HCC cell lines: PLC/PRF/5, Huh7 and HCC-LM3. Western blot analysis was performed to verify whether MTX1 was overexpressed in LV-MTX1 lentivirus infected cells (Figure 2A). Then cell growth was monitored by CCK-8 method. As shown in Figure 2B, MTX1 overexpression significantly promoted cell growth in the three cell lines. The similar results were also observed in colony formation assays (Figure 2C). Furthermore, we established a xenograft tumor model in nude mice to validate the role of MTX1 in tumorigenicity *in vivo*. HCC-LM3 cells with or without MTX1 overexpression were injected subcutaneously into nude mice. After four weeks, the tumor size and weight derived from MTX1 overexpressed cells were markedly larger and heavier than those derived from control group (Figure 2D). These data strongly suggested that MTX1 overexpression facilitates HCC cell growth and tumorigenicity *in vitro* and *in vivo*.

### MTX1 contributes to sorafenib resistance in HCC cells

**Figure 3:**
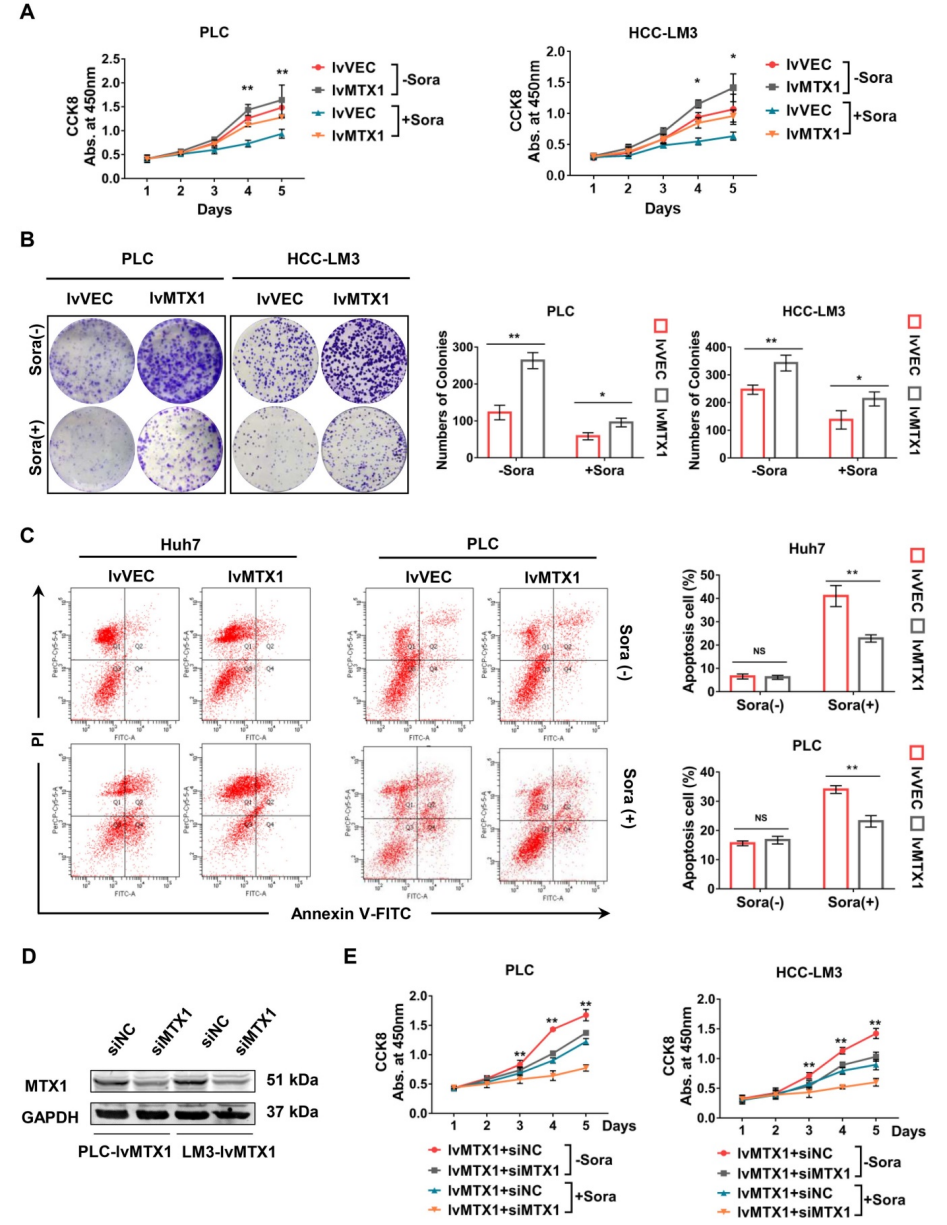
**MTX1 facilitated sorafenib resistance in HCC cells. (A)** Cell growth was assessed by CCK-8 assay in PLC/PRF/5 (lv-VEC, lv-MTX1) and HCC-LM3 (lv-VEC, lv-MTX1) cells with or without sorafenib treatment. **(B)** Clonogenic capacity of cells with enhanced MTX1 expression was detected with or without sorafenib treatment. **(C)** Flow cytometry was used to analyze the effect of MTX1 expression on cell apoptosis induced by sorafenib or DMSO treatment. **(D)** The knockdown efficiency of siMTX1 in MTX1-overexpressed PLC/PRF/5 and HCC-LM3 cells by western blotting. **(E)** Cell growth was examined by CCK-8 assay when MTX1 was knocked down in MTX1 overexpressed HCC cells. All above data are mean ± SD from three experiments. * P<0.05, ** P<0.01.

To investigate the association of MTX1 expression with HCC cell resistance to sorafenib, CCK-8 assay was used and the results indicated that MTX1 overexpression still maintained a higher cell growth rate compared with control cells under the treatment of sorafenib (Figure 3A). As the same time, the results from colony formation assays also exhibited a long-term resistance to sorafenib when MTX1 was overexpressed in HCC cells (Figure 3B). Furthermore, cell apoptosis was detected by flow cytometry under normal or sorafenib-treated condition. The results indicated that in non-sorafenib medium, the apoptosis rate of MTX1 overexpressed cells was not significantly different from that of the control cells, whereas MTX1 overexpressed cells showed an inhibition of apoptosis after sorafenib treatment (Figure 3C). To further validate the function of MTX1 in sorafenib resistance, a specific siRNA against MTX1 was synthesized. The interference efficiency was assessed by western blotting in PLC/PRF/5 and HCC-LM3 cells (Figure 3D). Cell growth analyses demonstrated that MTX1 knockdown significantly suppressed cell growth specifically at the presence of sorafenib (Figure 3E). These results implicated that MTX1 enhances sorafenib resistance and inhibition of MTX1 may be an effective approach for HCC therapy.

### High expression of MTX1 abrogates anti-tumor effect of sorafenib *in vivo*

**Figure 4:**
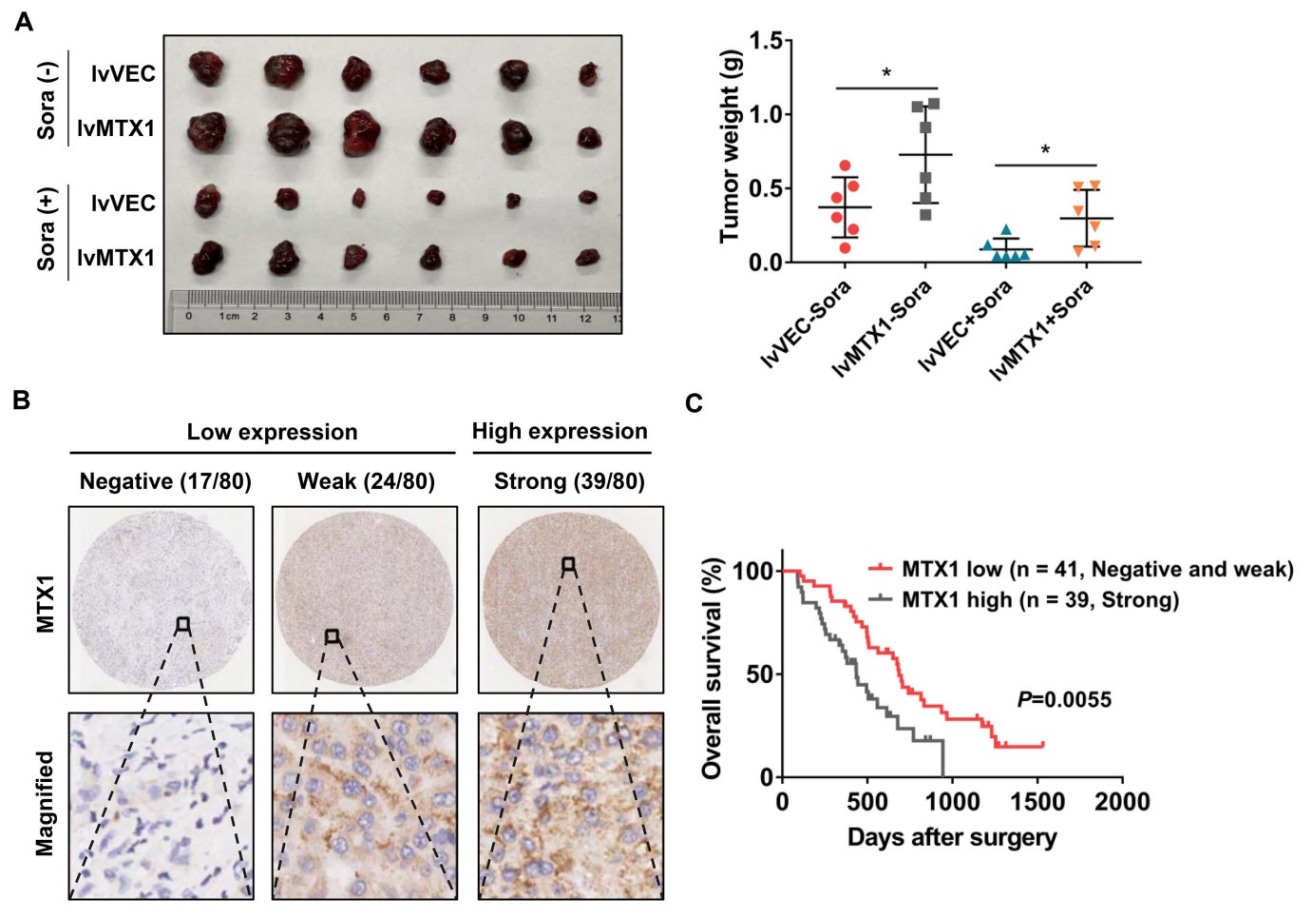
**MTX1 overexpression indicated anti-sorafenib effect in mouse model and HCC patients. (A)** The mice were subcutaneously injected with Huh7 (lv-VEC, lv-MTX1) cells and treated with DMSO or sorafenib by oral gavage. The tumor picture and tumor weight were shown after mice were sacrificed. **(B)** Representative immunohistochemical images displayed different levels of MTX1 expression in human HCC tissues. Scale bars, up: 200 μm; down: 50 μm. **(C)** Kaplan-Meier analysis were performed to compare the difference of overall survival rates between HCC patients with low and high MTX1 expression (n = 80). Data are mean ± SD from three independent experiments. * P<0.05.

To better understand whether MTX1 exerts sorafenib resistance role *in vivo*, Huh7 cells stably infected with MTX1 overexpression or control lentivirus were subcutaneously injected into the nude mice. One week later, these mice were randomly divided into two groups (n=6, each group) and then administered with control vehicle or sorafenib. When the largest tumor reached 1.5 cm in diameter, the mice were sacrificed and the weight of the tumor was measured. The final data clearly demonstrated that overexpression of MTX1 not only enhanced tumor formation ability, but also conferred resistance of HCC cells to sorafenib (Figure 4A). On the other hand, immunohistochemistry was performed on a tissue microarray containing 80 pairs of tumor tissues and normal tissues of HCC patients, who received sorafenib therapy after surgery. The staining results showed that MTX1 expression was high in 48.8%, weak in 30%, and negative in 21.2% of tumor cases according to the standard mentioned in methods section (Figure 4B). Additionally, by comparing overall survival of HCC patients in low and high MTX1 groups, we found that patients with high MTX1 expression had a worse prognosis after treatment with sorafenib (Figure 4C), suggesting that high expression of MTX1 in human HCC is involved in sorafenib resistance, consistent with the mouse experiment above.

### MTX1 enhances sorafenib resistance through promoting autophagy

**Figure 5:**
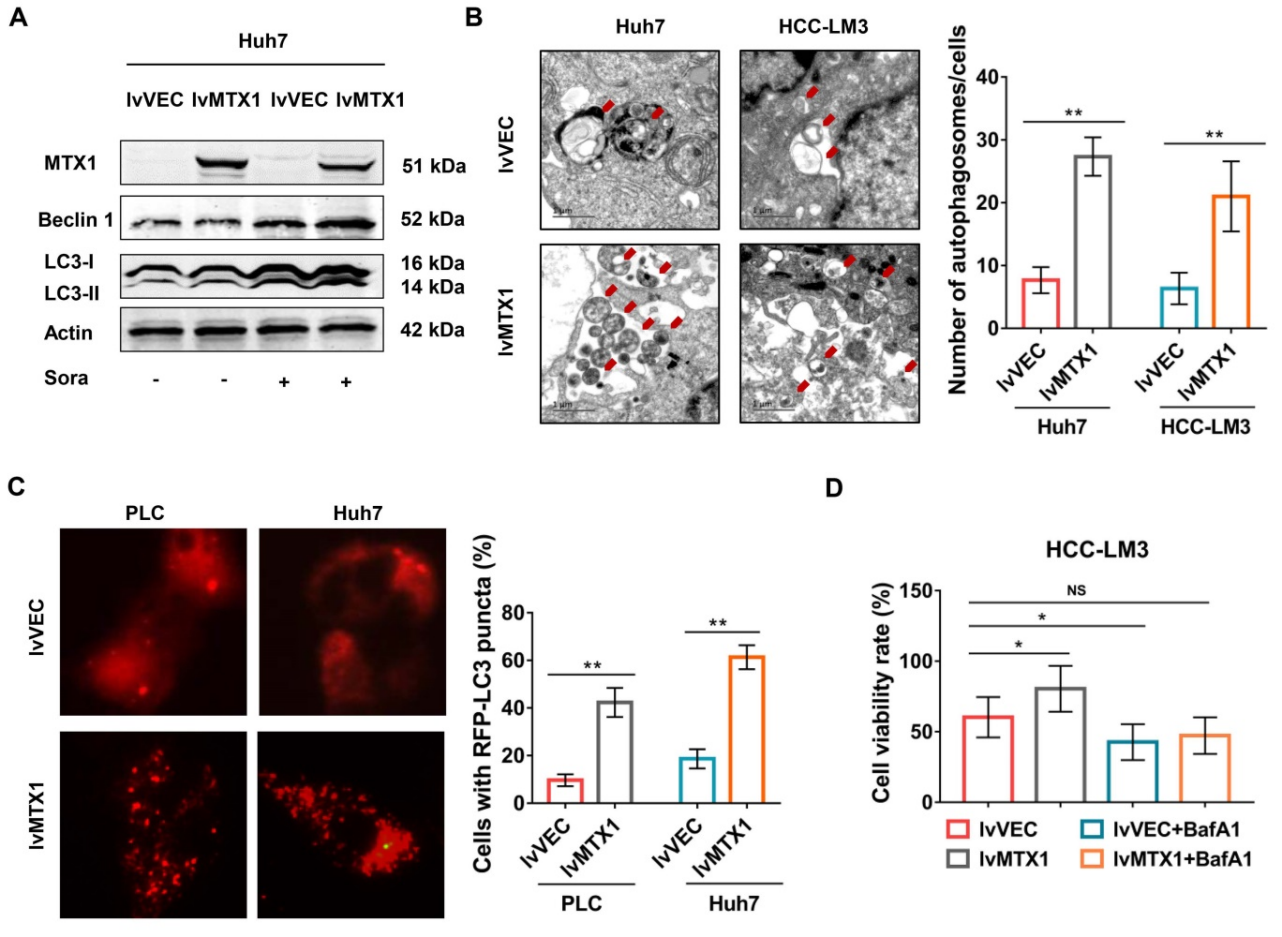
**MTX1 enhanced cell autophagy upon sorafenib treatment. (A)** The relative expression levels of autophagy-related genes were detected by western blot in Huh7 cells with or without MTX1 overexpression. **(B)** Autophagosomes were viewed under transmission electron microscope in MTX1 overexpressed or control cells after sorafenib treatment. Red arrows point to autophagosomes. Scale bars: 1 μm. **(C)** Representative fluorescent images of mCherry-LC3B in HCC cells with or without MTX1 overexpression after sorafenib cultivation were displayed, and the number of LC3-positive puncta in each cell was counted. **(D)** Growth curves of HCC-LM3 cells under -/+ BafA1 conditions are shown. All cells in this assay were treated with 5 μM sorafenib. Quantitative data are presented as mean ± SD. NS: no significance, * P<0.05, ** P<0.01.

Since autophagy is considered to be one of the major causes of sorafenib resistance 19-21, we next explored whether MTX1 affects autophagy of HCC cells. As expected, the protein levels of LC3 II and Beclin1 significantly upregulated in cells with MTX1 overexpression under sorafenib treatment conditions (Figure 5A). As the same time, electron microscopy examination indicated that autophagosomes were increased in Huh7 and HCC-LM3 cells with MTX1 overexpression compared with control cells after sorafenib treatment (Figure 5B). Moreover, the mCherry-LC3 puncta formation was observed by fluorescence microscope. MTX1 overexpression significantly increased the puncta formation in MTX1 overexpressed HCC cells upon sorafenib stimulation (Figure 5C). These results above strongly demonstrated that the MTX1 could induce autophagic response upon sorafenib treatment in HCC cells. To further investigate whether MTX1-mediated autophagy directly affects the sorafenib resistance of HCC cells, cell growth was assessed in the presence or absence of Bafilomycin A1 (BafA1), an autophagy inhibitor that blocks autophagic vacuole (AV)-lysosome fusion. The data showed that cell growth rate was significantly elevated along with MTX1 overexpression compared to the control group in the presence of sorafenib, while there was no difference between the cells treated with sorafenib and BafA1 (Figure 5D), indicating that MTX1-induced sorafenib resistance in HCC was eliminated through blocking the autophagic response.

### MTX1 promotes autophagy by interacting with CISD1

**Figure 6:**
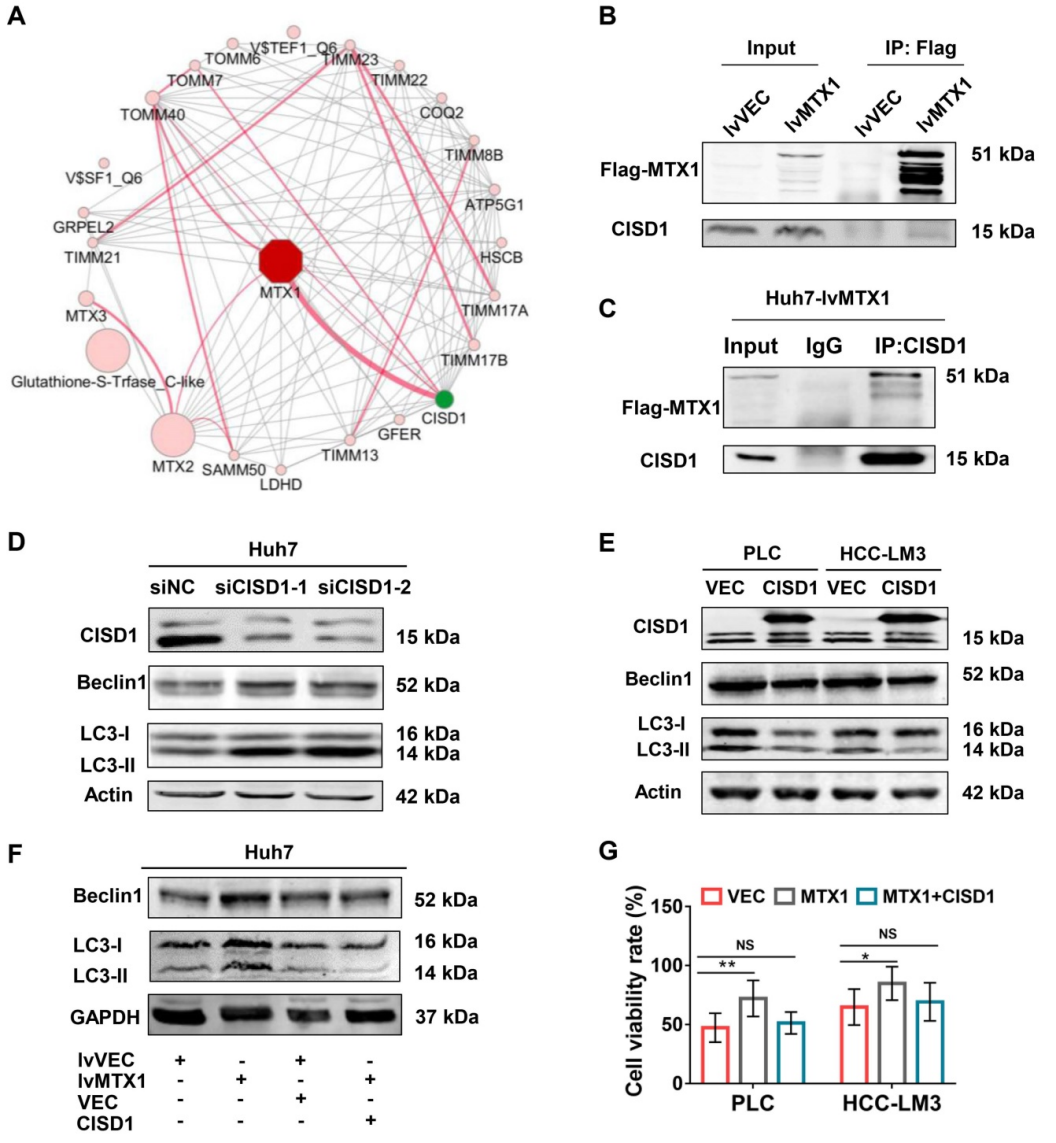
**MTX1 interacted with CISD1 to co-regulate HCC cell resistance to sorafenib. (A)** Protein interaction networks were obtained by GeneMANIA analysis performed on Cytoscape (Red lines). **(B)** Co-IP and western blot assays were performed using anti-Flag and Dynabeads Protein G to confirm semi-exogenous interaction between FLAG-MTX1 and CISD1. **(C)** Endogenous CISD1 antibody could pull down MTX1-FLAG protein through Co-IP assay. **(D, E)** Autophagy-related proteins were detected by western blot analysis when CISD1 was silenced or overexpressed. **(F)** CISD1 overexpression antagonized MTX1 overexpression-induced autophagy-related protein expression under the treatment of sofafenib. **(G)** CISD1 reduced cell viability induced by MTX1 overexpression. CCK-8 assay was used to monitor cell viability at 0 and 48 h after the treatment of sofafenib in HCC cells as indicated. Data are presented as mean ± SD from three experiments. NS: no significance, * P<0.05, ** P<0.01.

To address the mechanism by which MTX1 promotes autophagy, we used an online analysis tool GeneMANIA to predict proteins that interact with MTX1 in human. The protein-protein interaction network showed that CISD1, a known negative regulator of autophagy, was the most likely protein that interacts with MTX1 (Figure 6A). Co-immunoprecipitation (CoIP) experiments using anti-FLAG antibodies confirmed the association of FLAG-tagged MTX1 with CISD1 (Figure 6B). Reciprocally, endogenous CISD1 could also pull down the FLAG-tagged MTX1 in Huh7 cells (Figure 6C). To verify the role of CISD1 in the regulation of autophagy in HCC cells, western blot analyses were performed in HCC cells with CISD1 knockdown or overexpression. Consistent with another report in breast cancer^22^, CISD1 inhibited cell autophagy as determined by the changes of Beclin1 and LC3-II levels (Figure 6D, 6E). More importantly, the increase of Beclin1 and LC3-II induced by MTX overexpression could be impaired by CISD1, implying that the two proteins may play opposite function for cell autophagy. In addition, we attempted to investigate the influence of CISD1 on MTX1-mediated sorafenib resistance. CCK-8 assays indicated that CISD1 overexpression significantly abrogated the promoting effects of MTX1 on cell viability upon sorafenib treatment (Figure 6F). These findings significantly suggested that MTX1 affected the resistance of HCC cells to sorafenib via antagonizing CISD1-mediated suppression of cell autophagy.

## Discussion

Sorafenib, as a multi-targeted inhibitor, is an attractive drug for the treatment of HCC 23. However, most of patients develop uncontrollable progression because of drug resistance 24. In the current study, we identified MTX1 as a new regulator contributing to sorafenib resistance in HCC through CRISPR/Cas9 system screening. By evaluating the expression of MTX1 in HCC tissues via qRT-PCR and immunohistochemistry, we found that MTX1 is frequently overexpressed both in mRNA and protein levels. The immunohistochemical results also implicate the predictive significance of MTX1 in HCC for its high expression has a worse prognosis in patients with sorafenib treatment after excision survey. The functional investigations *in vitro* and *in vivo* confirmed that MTX1 plays a potential oncogenic role in HCC and the upregulation of MTX1 confers sorafenib resistance of HCC cells. This is the first time to report the function of MTX1 in cancer development and drug resistance to date.

MTX1 belongs to Metaxins family. The proteins in this family have long been considered to be responsible for transport of proteins into the mitochondrion 14, 25. A previous study has reported that MTX1 and MTX2 are required for mitochondrial transport in human neurons 26. Another report revealed that MTX1 and MTX2 were involved in Bak activation and tumor necrosis factor (TNF)-induced cell death 14, 15. Our findings illustrated the effect of MTX1 on promoting HCC progression and enhancing sorafenib resistance of HCC cells. As for the potential mechanism by which MTX1 participates in this process, we provided evidence that MTX1 could augment cell autophagy at the presence of sorafenib. In accordance with our data, a number of studies have indicated that some proteins or miRNAs, such as PSMD10, CD24, HANR and miR-375, are able to increase sorafenib resistance by regulating HCC cell autophagy 27-31. Our further explorations indicated that MTX1 interacts with CISD1 by Co-IP assays, whereas CISD1 functions as an autophagy repressor in cancer.

CISD1 is firstly identified in rodent brain, liver, and skeletal muscle 32, 33. As an outer mitochondrial membrane protein, CISD1 is associated with cellular oxidation process and plays an important role in regulation of ferroptosis and cellular respiration 32, 34-36. More importantly, it has been reported to contribute to breast cancer progression and suppress activation of autophagy 22. Its relationship with HCC progression has not been studied yet. From our present work, we can propose CISD1 and MTX1 may antagonize each other during cell autophagy via direct interaction. The functional analyses have revealed that overexpression of CISD1 attenuates MTX1 induced autophagy and cell growth rate upon sorafenib treatment, supporting our proposal. Further studies should be employed to provide a better understanding of the exact relationship between the two proteins in the future.

In addition to MTX1, our CRISPR/Cas9 screens also identified some other factors involved in sorafenib resistance, such as LENEP, BCL11B, BAG3 and RANBP1. These proteins may also be deserved to be studied in detail. Taken together, our present study identified MTX1 as an important factor contributing to HCC progression and sorafenib resistance via inducing cell autophagy. Targeting MTX1 might be a potential strategy for HCC therapy specifically combined with sorafenib.
